# Serum Vitamin D Levels Are Not Predictive of the Progression of Chronic Liver Disease in Hepatitis C Patients with Advanced Fibrosis

**DOI:** 10.1371/journal.pone.0027144

**Published:** 2012-02-16

**Authors:** Kathleen E. Corey, Hui Zheng, Jorge Mendez-Navarro, Aymin Delgado-Borrego, Jules L. Dienstag, Raymond T. Chung, the HALT-C Trial Group

**Affiliations:** 1 Gastrointestinal Unit, Massachusetts General Hospital, Boston, Massachusetts, United States of America; 2 Massachusetts General Hospital Biostatistics Center, Massachusetts General Hospital, Boston, Massachusetts, United States of America; 3 Division of Pediatric Gastroenterology, University of Miami, Miami, Florida, United States of America; 4 Department of Medicine, Harvard Medical School, Boston, Massachusetts, United States of America; Beijing Institute of Infectious Diseases, China

## Abstract

In animal models and human cross-sectional studies, vitamin D deficiency has been associated with liver disease progression. Vitamin D supplementation has been suggested as a treatment to prevent disease progression. We sought to evaluate the role of vitamin D levels in predicting chronic liver disease development. We conducted a nested case-control study of vitamin D levels in subjects with (cases) and without (controls) liver histologic progression or clinical decompensation over the course of the HALT-C Trial. Vitamin D levels were measured at 4 points over 45 months. 129 cases and 129 aged-matched controls were included. No difference in baseline vitamin D levels were found between cases and controls. (44.8 ng/mL vs. 44.0 ng/mL, P = 0.74). Vitamin D levels declined in cases and controls over time (P = 0.0005), however, there was no difference in the level of decline (P = 0.37). Among study subjects with diabetes mellitius, baseline vitamin D levels were higher in cases, 49.9 ng/mL, than controls, 36.3 ng/mL. (P = 0.03) In addition, baseline vitamin D levels were higher in black case subjects, 32.7 ng/mL, than in black control subjects, 25.2 ng/mL (P = 0.08) No difference in vitamin D levels was found between patients with and without progression of hepatitis C-associated liver disease over 4 years. Our data do not suggest any role for vitamin D supplementation in patients with advanced chronic hepatitis C and raise the possibility that higher vitamin D levels may be associated with disease progression.

## Introduction

Hepatic fibrosis results from wound healing following acute and chronic liver injury. In response to chronic hepatic inflammation, parenchymal cells release extracellular matrix proteins including type I collagen, resulting in the progressive deposition of, and accumulation of, fibrosis. Ultimately, fibrotic tissue can replace hepatocytes and disrupt lobular architecture, the hallmark of cirrhosis, which, in turn, results eventually in hepatic dysfunction. [1]

Emerging data suggest that vitamin D is an important modulator of both the inflammatory response and wound healing. [2], [3] Vitamin D may modulate the inflammatory response and subsequent fibrosis via inhibition of TNF-α, a cytokine that plays a central role in the regulation of the immune response [4], [5] and by inhibiting the development of fibrosis directly through suppression of TGF-β, a multifunctional cytokine that may influence fibrosis progression [6].[7].[8].

The importance of vitamin D in immune modulation and deposition of fibrosis may extend to the liver, which plays an important role in vitamin D homeostasis. The liver is the site of the conversion of vitamin D3 to 25-hydroxy-vitamin D (25-OH-vitamin D) and may be a site of vitamin D storage. [9] In addition, vitamin D receptors exist on hepatocytes and other hepatic parenchymal cells, including hepatic stellate cells. As in the kidney, vitamin D is postulated to play an antiinflammatory and antifibrotic role in the liver via binding to promoters of target genes, leading to down-regulation of TNF-α and TGF-β production.

Cross-sectional population data suggest that vitamin D deficiency is common in persons with advanced liver disease. [10], [11] For example, Fisher et al. [12] evaluated vitamin D levels in 100 patients with liver disease, 51 with cirrhosis and 49 without cirrhosis, including 38 patients with chronic hepatitis C. The prevalence of vitamin D deficiency was significantly higher in cirrhotic than noncirrhotic subjects (86.3% versus 49.0%, p = 0.0001). Moreover, vitamin D levels decreased with advancing Child class; vitamin D levels were significantly lower in subjects with Child class C (22.7 nmol/L) than in those with Child class A (45.8 nmol/L, p<0.001). Such studies, however, are limited by their cross-sectional nature. Thus, while an association between vitamin D deficiency and advancing liver disease has been noted, the potential that vitamin D deficiency could be a predictor for progressive liver disease has not been explored.

Recently, vitamin D has been evaluated as a potential immunomodulator of hepatitis C virus (HCV), and preliminary data suggest that the addition of vitamin D to standard antiviral therapy may improve treatment response rates. [13] This observation lends further weight to the potential immunomodulatory role of vitamin D in liver disease.

The progression of hepatic fibrosis occurs over years, hampering the study of processes that affect fibrosis. On the other hand, the Hepatitis C Antiviral Long-term Treatment against Cirrhosis (HALT-C) Trial provided a unique opportunity to study fibrosis longitudinally over several years. [14] The HALT-C Trial was a 10-clinical center randomized, controlled trial to evaluate the benefit of long-term (3.5-years) peginterferon therapy in patients with histologically advanced (Ishak fibrosis stage ≥3) but clinically compensated chronic hepatitis C who had failed to respond previously to antiviral therapy. While the HALT-C Trial was a negative study, showing that such maintenance antiviral therapy was ineffective, the trial's study design, including sequential liver biopsies spanning 4 years rendered the trial population ideally suited for the study factors that might influence fibrosis progression. Therefore, we conducted a nested case-control study of vitamin D levels in subjects with and without liver histologic progression or clinical decompensation over the course of the HALT-C Trial.

## Results

### Baseline Characteristics of Cases and Controls

One hundred twenty-nine cases met the inclusion criteria and 129 aged matched controls were selected. (Table 1). Cases with progression were defined (as the primary outcomes in the HALT-C Trial) as subjects with Ishak fibrosis stage 3 or 4 who experienced (1) progression of fibrosis over the study period, defined by an increase in Ishak fibrosis score of ≥2 stages, (2) an increase in the Child-Turcotte-Pugh (CTP) score to ≥7 on two successive study visits (3 months apart), (3) hepatic decompensation, defined by the presence of ascites, hepatic encephalopathy, variceal hemorrhage, spontaneous bacterial peritonitis, or (4) death. Controls were subjects with stage 3 or 4 fibrosis who did not experience any of the primary study endpoints by the end of 4-years in the trial (a 24-week lead-in phase when all participants received full-dose peginterferon alfa-2a and weight-based ribavirin and a 3-½-year randomized phase of half-dose maintenance peginterferon therapy or observation).

**Table 1:** Baseline characteristics of the cases and controls in the nested analysis.

	Case N (%)	Controls N (%)	P Value
N	129	129	
Male (%)	89 (69.0)	100 (77.5)	0.16
Female (%)	40 (31.0)	29 (22.5)	
Non-Hispanic White (%)	99 (76.7)	91 (70.5)	0.34
Black (%)	19 (14.7)	27 (20.9)	
Hispanic (%)	8 (6.2)	5 (3.9)	
Other (%)	3 (2.3)	6 (4.7)	
Mean Age (± SD)	49.5±6.5	50.0±7.3	0.54
Mean BMI (± SD)	30.5±5.4	29.5±5.0	0.16
BMI Profile (%)			0.19
- <18.5	0(0)	1(0.78%)	
- 18.5–24.9	14(10.85%)	20(15.50%)	
- 25–29.9	52(40.31%)	62(48.06%)	
- 30–34.9	48(37.21%)	31(24.03%)	
- 35–39.9	10(7.75%)	8(6.20%)	
- >40	5(3.88%)	7(5.43%)	
HCV Genotype 1	119(92.3)	124 (96.1)	0.28
HCV Genotype other	10 (7.8)	5 (3.9)	
HCV RNA Log_10_ IU/ml (± SD)	6.4±0.5	6.5±0.5	0.33
Mean Ishak Histologic Activity Index (± SD)	7.4±2.1	7.0±1.9	0.15
ALT (U/L)	136.4±125.7	103.8±77.6	0.013
Steatosis (%)			0.002
0	20(15.50%)	28(21.71%)	
1	38(29.46%)	62(48.06%)	
2	46(35.66%)	28(21.71%)	
3	21(16.28%)	10(7.75%)	
4	4(3.10%)	1(0.78%)	
Mean Glucose mg/dL (+/−SD)	113±46	105±35	0.12
Mean Insulin (+/−SD)	54.6±39.7	43.1±45.4	0.06
Mean HOMA-IR	16.8±16.6	12.6±17.7	0.09
Average Number of drinks per year (± SD)	683.7±1056.2	641.2±875.0	0.73
Average grams of ETOH per day (± SD)	22.5±34.7	21.1±28.7	0.73
Diabetes (%)	27 (20.9)	16 (12.4)	0.09
Pegylated Interferon Treatment	60 (46.5)	65 (50.4)	0.53
Blood drawn during winter months (out of 1023 draws)	284 (48.8)	298 (51.2)	0.44

The mean age was 49.5 years in the cases and 50.0 years in the controls, the mean BMI was 30.5 in the cases and 29.5 in the controls (P = 0.16), genotype 1 was predominant in both groups, and the mean viral load was 6.4 and 6.5 log_10_ IU/ml in cases and controls, respectively. Histologic Activity Index did not differ significantly between the two groups (P = 0.15). However, the cases had a significantly higher baseline ALT levels than controls (136.4 versus 103.8, P = 0.013). In addition, cases had more steatosis than controls (p = 0.002). 50.4% of control patients and 46.5% of case patients received pegylated interferon during the HALT-C trial (P = 0.53).

To further ensure that our cases and controls were appropriately selected we analyzed end of study levels of albumin, prothrombin time and platelets. Cases had significantly lower platelets than control patients (131.1 vs 180.7, p<0.0001) and significantly lower albumin level (3.77 vs 4.06, p<0.0001) and higher prothrombin times (1.09 vs 1.03, p<0.0001) suggesting that cases were correctly classified as having progressive disease.

When evaluated by HALT-C treatment status (pegylated interferon versus placebo) there was no difference in the distribution of gender, ethnicity, age, BMI, steatosis, HAI, glucose, HOMA-IR, ALT, genotype, average drinks per day, prevalence of diabetes, albumin, platelet count, total bilirubin. Subjects who received pegylated interferon had statistically lower baseline HCV RNA log (6.19 IU/mL vs 6.42 IU/mlL, p = 0.01).

Over the duration of the HALT-C Trial, 97.7% (n = 126) of cases had a ≥2-point increase in Ishak fibrosis score, and 17.1% (n = 22) had an increase in CTP score to ≥7. Encephalopathy developed in 6.2% (n = 8) of cases, ascites in 7.8% (n = 10), variceal hemorrhage in 2.3% (n = 3), and spontaneous bacterial peritonitis in 1.6% (n = 2) of patients. The death rate among cases was 5.4% (n = 7) and 3.1% among controls (n = 4), which was not statistically different (p = 0.54).

### Factors Associated with Vitamin D Deficiency

Two vitamin D measurements were made in this study, vitamin D3 and vitamin D2 levels. Vitamin D3 levels reflect endogenous vitamin D while vitamin D2 levels reflect exogenous vitamin D (or vitamin from supplements). Total vitamin D is the sum of the vitamin D2 and Vitamin D3 levels.

Vitamin D levels have been shown to vary by latitude and season with higher vitamin D levels found in summer months and at latitudes closer to the equator. [15] Thus, we evaluated whether our cases and controls differed by latitude of HALT-C site and season during which blood was drawn.

We found no difference in the months of blood draws between cases and controls (P = 0.53) or in the season when evaluating winter (October to April) compared to summer (May to September) (P = 0.44). In addition, there was no difference in the location of clinical center between cases and controls. In the control group 27.8% of subjects and 27.1% of subjects in the case group came from latitudes of 35 degrees or less compared to 72.2% at latitudes greater than or equal to 35 degrees in the controls and 72.9% of cases. (P = 0.91)

In addition, we evaluated the difference in vitamin D levels based on HALT-C treatment status. No significant difference in vitamin D level was seen between subjects who received pegylated interferon and those who received placebo (34.7 mg/dL vs 35.7 mg/dl, p = 0.61). When further divided by cases and controls there was again no difference between subjects who received pegylated interferon and those who received placebo. Among cases who received pegylated interferon, mean vitamin D level was 34.7 mg/dL compared to 36.4 mg/dL in the placebo group (p = 0.52). Among controls who received pegylated interferon mean vitamin D level was 34.7 mg/dL compared to 35.0 mg/dL in the pacebo group p = 093).

A low vitamin D level was associated independently with black race (P<0.0001). (Table 2) Vitamin D levels were also significantly associated with the month of blood draw (P<0.0001). These remained significant when adjusted for the age, gender, BMI, HCV RNA level, diabetes, HAI, race, site of draw, month of draw and genotype. Clinical trial site was associated with vitamin D level with sites at latitudes of 35 degrees or below having significantly higher vitamin D levels than those above 35 degrees (40.1 mg/dL vs 46.0 mg/dL, P = 0.02) but was not significant when adjusted for the remaining variables. Vitamin D level was also found to be higher in patients with genotype 1 compared to non-genotype 1 patients (+10.75 mg/dL, p = 0.04) but is limited by only 15 patients being non-genotype 1. This association was not significant on multivariate analysis (p = 0.09) Vitamin D levels were not related to age, gender, BMI, HCV RNA level, presence of diabetes mellitus, Histologic Activity Index, or ALT when multivariate analysis was performed.

**Table 2:** Factors associated with vitamin D level (univariate).

Baseline Characteristics	P Value
HCV RNA level	0.45
BMI	0.73
Age	0.06
Gender	0.89
ALT U/L	0.61
Diabetes mellitus	0.81
Average alcoholic drinks per year	0.13
Race	0.0001
HCV Genotype	0.04
Diabetes or blood sugar >126 mg/dL	0.73
Month of blood draw	<0.0001
Site of blood draw	0.006

### Are Vitamin D Levels Predictive of the Progression of Chronic Liver Disease?

Vitamin D levels were assessed at the time of HALT-C Trial screening, randomization to peginterferon or placebo (week 24), at month 27, and month 45 (Table 3). Baseline total vitamin D levels were 44.8 ng/mL in the cases and 44.0 ng/mL in the controls (P = 0.74). Levels in both groups declined over the study period, falling to 44.4 ng/mL in cases and in 44.2 ng/mL controls at the time of randomization (P = 0.91), to 40.5 ng/mL in cases and 39.2 ng/mL in controls at month 27 (P = 0.59), and to 38.8 ng/mL in cases and 40.9 ng/mL in controls at month 45 (P = 0.32). While vitamin D levels declined significantly in both cases and controls over time (P = 0.0011), no difference emerged in the level of the decline between the case and control groups (−5.6 ng/mL versus −3.3 ng/mL respectively, P = 0.77). In addition, the levels of vitamin D2 (exogenous vitamin D derived from supplement use) and vitamin D3 (endogenous) did not differ between cases and controls at any time point during the study.

**Table 3:** Mean vitamin D (ng/mL) levels in cases and controls.

	Cases (n = 129)			Control (n = 129)			P value		
	Total	D2	D3	Total	D2	D3	Total	D2	D3
**Month 0 (Screening)**	44.8±19.4	3.0±6.1	41.6±18.3	44.0±19.7	2.5±4.7	41.4±19.9	0.74	0.47	0.94
**Week 24 (Randomization)**	44.4±19.8	3.1±5.8	41.4±20.2	44.2±19.7	2.2±4.3	41.2±19.0	0.91	0.17	0.92
**Month 27**	40.5±17.3	3.2±5.9	37.6±17.4	39.2±20.7	2.3±4.3	35.6±17.6	0.59	0.15	0.38
**Month 45**	38.8±18.4	3.2±5.6	35.6±18.3	40.9±13.8	3.4±5.7	37.6±14.1	0.32	0.87	0.33

The prevalence of vitamin D deficiency, defined as is standard by a vitamin D level ≤30 ng/ml, was indistinguishable between cases, 24.6%, and controls, 23.8% (P = 0.88). Similarly, extreme vitamin D deficiency, defined by a vitamin D level ≤20 ng/mL, was found in 7.1% of cases and 10.4% of controls (P = 0.38).

We suspected that while vitamin D levels may not be significantly different in our cohort as a whole, vitamin D deficiency may be higher in cases and controls in certain subgroups, diabetic subjects and black subjects, at higher risk for vitamin D deficiency. We found that in subjects with an established diagnoses of diabetes mellitius or an elevated fasting glucose >126, baseline vitamin D levels were higher in cases, 47.9 ng/mL, than controls, 36.3 ng/mL (P = 0.03) (Table 4 and 5). In addition, baseline vitamin D levels were higher in black subjects who had progressive liver disease, 32.7 ng/mL, than in black control subjects, 25.2 ng/mL (P = 0.08) (Table 6). These findings suggest, contrary to our belief, that increased serum vitamin D levels may be associated with a risk of liver-disease progression in specific groups with other risk factors.

**Table 4:** Baseline characteristics of diabetic cases and controls in the nested analysis.

	Case N (%)	Controls N (%)	P Value
N	27	16	
Male (%)	20(74%)	15(94%)	0.11
Female (%)	7(26%)	1(6%)	
Non-Hispanic White (%)	20(74.07%)	6(37.50%)	0.04
Black (%)	5(18.52%)	8(50%)	
Hispanic (%)	1(3.70%)	2(12.50%)	
Other (%)	1(3.70%)	0(0%)	
Mean Age (± SD)	52±7.0	52±6.4	0.88
BMI Profile (%)			0.55
- <24.9	2(7.41%)	4(25%)	
- 25–29.9	11(40.74%)	7(43.75%)	
- 30–34.9	9(33.33%)	3(18.75%)	
- 35–39.9	2(7.41%)	1(6.25)	
- >40	3(11.11)	1(6.25)	
Mean Ishak Histologic Activity Index (± SD)	6.44±1.65	7.25±1.61	0.12
ALT (U/L)	103.7±67.9	84.1±51.7	0.33
Steatosis (%)			
0	6(22.22%)	6(37.50%)	0.34
1	9(33.33%)	8(50%)	
2	7(25.93%)	1(6.25%)	
3	4(14.81%)	1(6.25%)	
4	1(3.70%)	0(0%)	
Average Number of drinks per year (± SD)	653±758	652±823	0.99

**Table 5:** Mean baseline vitamin D (ng/mL) levels by in patients by diabetes status.

	Cases	Controls	P value
**Diabetes**	47.9±19.4	36.3±16.0	P = 0.03
**Non-DM**	43.7±19.4	45.4±20.1	P = 0.53

**Table 6:** Mean baseline vitamin D (ng/mL) levels by race.

	Cases	Controls	P Value
**African Americans**	32.7±15.8	25.2±12.4	0.08
**White**	47.2±19.7	49.7±18.5	0.36
**Hispanic**	48.8±13.6	53.8±15.2	0.54
**Other**	32.1±17.1	32.0±5.6	0.99

### Vitamin D Supplement Use

The self-reported use of vitamin D supplements was not available from the HALT-C study. However, vitamin D supplement use was directly measured by assaying for vitamin D2 levels. Over the course of the four year study 118 subjects did not have a detectable vitamin D2 level at any of the four time points indicating no supplement use with the remainder having a detectable vitamin D2 level at at least one time point. There was no significant difference in this distribution between cases and controls. (P = 0.14).

We analyzed the mean total vitamin D levels, vitamin D3 and vitamin D2 levels in those subjects with detectable vitamin D2 levels on all four occasions to determine if supplement use was greater in control patients and could account for a lack of progression of chronic liver disease (n = 28). We noted that at baseline cases had significantly higher vitamin D3 levels (46.3 mg/dL vs 32.4 mg/dL, P = 0.03) and total vitamin D levels (60.0 mg/dL vs 43.3 mg/dL, P = 0.02) when compared to control patients. (Table 7) Vitamin D2 levels, indicative of supplement use, had a trend toward higher levels in cases compared to controls (13.7 mg/dL vs 10.9 mg/dL, P = 0.28) although this did not reach statistical significance. We did not find, however, the supplement use or higher total vitamin D levels were associated with improved outcomes and our findings suggest that higher vitamin D levels may be associated with progression of liver disease.

**Table 7:** Analysis of vitamin D levels in patients with detectable vitamin D2 at all time points.

	Cases (n = 16)			Control (n = 12)			P value		
	Total	D2	D3	Total	D2	D3	Total	D2	D3
**Month 0 (Screening)**	60.0±15.9	13.7±6.6	46.3±15.7	43.3±19.0	10.9±6.6	32.4±17.2	0.02	0.28	0.03
**Week 24 (Randomization)**	44.5±14.0	11.8±5.7	32.7±12.2	50.8±15.2	9.5±5.7	41.3±16.4	0.27	0.30	0.12
**Month 27**	41.6±12.9	12.3±5.3	30.7±10.7	39.0±7.9	9.4±3.7	29.6±8.5	0.54	0.12	0.77
**Month 45**	41.6±14.6	11.9±6.2	29.7±12.7	43.3±13.8	10.7±5.6	32.7±13.7	0.75	0.60	0.57

This is publication #59 of the HALT-C Trial.

Further, we analyzed the baseline vitamin D levels in patients who were taking vitamin D supplements at the time of enrollment (N = 77). We found that total vitamin D levels were significantly higher in the cases (53.6 mg/dL) when compared to controls (44.7 mg/dL, P = 0.04). Cases had a non-significant increase in both vitamin D2 (10.5 mg/dL vs. 8.1 mg/dL, P = 0.10) and vitamin D3 levels (42.5 mg/dL vs. 36.6 mg/dL, P = 0.16) when compared to controls.

## Discussion

In this study, we had the opportunity to evaluate longitudinally the impact of vitamin D levels on the progression of chronic liver disease. Our nested case control study suggests that vitamin D levels do not influence the progression of chronic liver disease. We found no difference in mean vitamin D levels in patients with and without progressive chronic liver disease during any point over 45 months. Vitamin D levels declined over time in both groups consistent with the known effect of aging on vitamin D levels but persons with progression of liver disease did not experience a greater decline than persons without disease progression. [16] The existing literature contains conflicting evidence on the relationship between vitamin D and chronic liver disease. Our findings are supported by several studies in the literature evaluating vitamin D levels in chronic viral liver disease. Duarte et al evaluated 100 persons with chronic hepatitis C and found no difference in mean vitamin D levels in those with and without cirrhosis. Their levels of vitamin D, 46.6 ng/mL in non-cirrhotic persons and 45.6 ng/mL in cirrhotic patients (P = NS), were similar to the levels in our study. [17] Gallego-Rojo et al found similar levels with Child's Class A cirrhotic patients having a non-significant increase in vitamin D when compared to healthy controls (48.1 ng/mL vs. 45.5 ng/mL, respectively). [18] However, in several studies decreasing vitamin D levels have been associated with progression of chronic liver disease. [10], [12], [19], [20]. However, these studies have several important limitations in their design that may explain the different findings. First, these studies are universally cross sectional in nature, capturing the relationship between vitamin D and liver disease at only a single point in time and therefore are unable to assess changes in vitamin D temporally with the progression of liver disease. [10], [12], [19], [20]. In addition, control subjects have in large part consisted of healthy controls introducing potentially confounders. Vitamin D levels have been found to be decreased in persons with a number of chronic diseases including hypertension, diabetes mellitus, nephritic syndrome and chronic kidney disease and further vitamin D level is considered an excellent marker of overall general health. [21], [22] Thus, vitamin D deficiency may be the result of the chronic disease state rather than specifically the result of chronic liver disease which would not be highlighted by the use of healthy controls. Our study design, however, allows for controlling for the presence of chronic disease by choosing controls with chronic liver disease. By controlling for chronic disease we found that no difference was apparent in vitamin D levels.

Several advantages exist in the design of our study when compared to previously published works on vitamin D in chronic liver disease. First, our study was a nested case control rather than a traditional case control study. Nested case control studies have a distinct advantage from traditional case control studies because both cases and controls are chosen from the same, well-defined source population, in this study from the HALT-C study population where all patients had chronic hepatitis C infection with chronic liver disease. This is in contrast to other recently published works of cross-sectional case control studies where controls were uninfected healthy subjects. [20] Further, the traditional disadvantage of nested case control studies, which the cases and controls differ due to a higher death rate or loss to follow-up in the controls was not seen in HALT-C where no patients were lost to follow-up and only 21 deaths occurred.

In this study we also found that in specific subgroups, diabetic patients and black patients, cases had higher vitamin D levels than controls suggesting that elevated levels of vitamin D may be harmful in these subgroups. Diabetic and black patients may be more susceptible to fibrosis progression, a progression that may be exacerbated by elevated levels of vitamin D. [20], [23], [24], [25] While our study was not designed to evaluate the effect of vitamin D on fibrosis in these specific subgroups further study the suggestion of an association between elevated levels of vitamin D and accelerated disease progression in these groups warrants further evaluation.

Our study has several important limitations. First, in three quarters of our study subjects, the mean vitamin D levels were normal. This high proportion of normal vitamin D levels may be the result of the high supplement use among this group. Over the course of this study 140 subjects (54.7%) had detectable vitamin D2 levels, evidence of supplement use. In addition, subjects willing to participate in this rigorous long term trial may be highly motivated and have increased outdoor exercise and sun exposure leading to higher vitamin D levels. If the benefit of vitamin D were limited to vitamin D-deficient subjects, our study would have been underpowered to detect such an effect of vitamin D. On the other hand, in the two subgroups that did show a difference in vitamin D levels between cases and control, diabetics and black patients, vitamin D levels were higher in cases with histologic and/or clinical progression than in stable controls. Furthermore, in black subjects, mean vitamin D levels were in the deficiency range (mean 28.6 ng/mL); yet, vitamin D levels were higher in cases and then in controls.

In addition, our study is limited by its evaluation of only patients in the HALT-C study. The HALT-C study was limited to subjects who did not achieve sustained virologic response (SVR) to standard therapy. Low SVR rates have been associated with low vitamin D levels and suggest that at interaction between normal or high vitamin D levels may impact SVR. The patients in HALT-C, non-responders to therapy, may be a select group who do not benefit from normal or increased vitamin D levels and whose disease progression is not impacted by vitamin D.

In addition, in our study, we limited our evaluation to patients with Ishak fibrosis stage 3–4. Potentially, the benefits of vitamin D elevation (and supplementation) are negligible once this degree of fibrosis has occurred; our study group would not have revealed whether vitamin D may be beneficial in patients with no or early-stage fibrosis. On the contrary, by focusing on patients with stage 3–4 hepatic fibrosis, we were targeting patients with a high risk of disease progression, a group in which we would have expected to see the greatest potential benefit. In addition, we evaluated only patients with hepatitis C-induced liver disease and did not evaluate the role of vitamin D in other forms of chronic liver disease. Potentially, the impact of vitamin D levels in other types of chronic liver disease might be different.

Finally, while the HALT-C study had more than 4 years of follow-up this may still be insufficient follow-up time to adequately assess our desired outcomes. As was recently seen in an extended cohort of HALT-C, maintenance interferon therapy was associated with a reduced risk of HCC in cirrhotic patients. [26] Thus, vitamin D may be a predictor of longer term outcomes that cannot be assessed in the original HALT-C study.

In conclusion, we found no difference in vitamin D levels between patients with and without progression of hepatitis C-associated chronic liver disease over the course of nearly 4 years. Although we have not conducted a randomized trial of vitamin-D supplementation, our data do not suggest any role for vitamin D supplementation in patients with histologically advanced chronic hepatitis C and even raise the possibility that vitamin D supplementation may be harmful. Of course, vitamin D supplementation has been linked with a myriad of other health benefits, and our data are insufficient to support withholding vitamin D from these patients. On the side of caution, however, further study is needed in to evaluate the role of vitamin D supplementation in patients with chronic liver disease who are diabetic or black.

## Methods

This ancillary study was approved by the Partners Human Research Committee. The HALT-C Trial was approved by institutional review boards at each of the participating sites, and all study subjects provided written informed consent. Participating sites included University of Massachusetts Medical School, Worcester, MA; University of Connecticut Health Center, Farmington, CT; Saint Louis University, Saint Louis, MO, Partners Healthcare, Boston, MA; University of Colorado Health Sciences, Aurora, CO; University of California, Irvine, Ivine, CA; Long Beach VAMC Research Health Care Group, Long Beach, California; University of Texas Southwestern Medical Center, Dallas, TX; University of Southern California Health Sciences Campus, Los Angeles, CA; University of Michigan Medical School, Ann Arbor, MI; Virginia Commonwealth University, Richmond, VA; National Institute of Diabetes and Digestive and Kidney Diseases National Institutes of Health, Bethesda, MD.

### Study Design

This was a case-control study to evaluate the association of serum 25-OH-vitamin D levels with fibrosis progression. Cases with progression were defined (as the primary outcomes in the HALT-C Trial) as subjects with Ishak fibrosis stage 3 or 4 who experienced (1) progression of fibrosis over the study period, defined by an increase in Ishak fibrosis score of ≥2 stages, (2) an increase in the Child-Turcotte-Pugh (CTP) score to ≥7 on two successive study visits (3 months apart), (3) hepatic decompensation, defined by the presence of ascites, hepatic encephalopathy, variceal hemorrhage, spontaneous bacterial peritonitis, or (4) death. Controls were subjects with stage 3 or 4 fibrosis who did not experience any of the primary study endpoints by the end of 4-years in the trial (a 24-week lead-in phase when all participants received full-dose peginterferon alfa-2a and weight-based ribavirin and a 3-½-year randomized phase of half-dose maintenance peginterferon therapy or observation). We chose to expand our primary endpoint beyond fibrosis progression alone to limit the impact of the sampling variability of needle-liver biopsy as well as to include clinically significant outcomes. Controls and cases were matched for age (age less than 49 years of age or age equal to or greater than 49 years of age) Because hepatocellular carcinoma (HCC) may have independent effects on vitamin D homeostasis, we excluded patients in whom HCC developed. [27]

### Inclusion Critieria

To be included as a case or a control in the nested case-control study, HALT-C Trial participants had to have (1) liver histopathology available at study entry and study cessation (baseline and 4 years [biopsies were done at baseline, year 2 and year 4]), (2) stored serum available for 25-OH-vitamin D assay, and (3) Ishak fibrosis stage 3 or 4 at study entry. Subjects with Ishak Fibrosis stage 5 or 6 at study entry, absence of serial liver biopsies, and those in whom HCC developed during the study period were excluded. Two hundred fifty-eight subjects from the HALT-C Trial met these inclusion criteria including 129 cases and 129 controls.

### Endpoints

The primary endpoint was the mean vitamin D level at study entry; secondary endpoints were vitamin D levels at randomization (trial-week 24), month 27, and month 45. –Total 25-OH-Vitamin D was calculated from the sum of 25-OH vitamin D3 (endogenously produced vitamin D) and 25-OH-vitamin D2 (derived from supplements). Serum samples were aliquoted and frozen immediately at −70°C at each of the 10 clinical centers, then shipped on dry ice and stored at a central contract repository site. For the measurement of vitamin D levels deuterated stable isotope [d3-25-hydroxyvitamin D] was added to a 200 uL serum specimen as an internal standard. The specimen was then deproteinated by acetonitrile precipitation. 25-hydroxyvitamin D2, 25-hydroxyvitamin D3, and internal standard in the organic supernate were purified by a liquid chromatography system. Purified hydroxyvitamin D2, 25-hydroxyvitamin D3, and internal standard are ionized at atmospheric pressure and injected into a tandem mass spectrometer and quantified relative to calibrators prepared in charcoal-stripped human serum. The limit of quantitation for 25-hydroxyvitamin D2 is 2 ng/mL [CV = 15%] and for 25-hydroxyvitamin D3 is 3 ng/mL [CV = 25%]. The between-run CV for a quality control serum containing a total vitamin D concentration of 23 ng/mL is 7.5%. Storage time, up to 24 years, has shown to have no effect on vitamin D levels in stored serum. [28]

### Statistical Analysis

We evaluated serum vitamin D levels in cases and controls as continuous variables. Baseline was defined as time of randomization. For those patients whose information was missing at randomization, information at screening was used as baseline. The cases and controls were weakly matched by age only. As a result, the correlation between case and control were very weak and the two groups can be considered independent. Student's t test or Wilcoxon rank sum test was used as appropriate. Fisher's exact test or Chi-square test was used for bivariate analyses of categorical variables whenever appropriate. In addition, we evaluated serum 25-OH-vitamin D levels as categorical variables (binary: deficient and normal value as well as in quartiles) with the chi square test to create an odds ratio for the progression of fibrosis based on vitamin D levels. We performed multivariate modeling to evaluate the impact of vitamin D levels as well as other known variables that influence fibrosis (age, estimated duration of HCV infection, body mass index [BMI], diabetes mellitus, hypertension, alcohol use) on fibrosis progression. In addition, we performed a linear regression analysis to evaluate the impact of vitamin D levels on HCV RNA levels. With a sample size of 129 subjects per study arm, we had an 80% power to detect a mean difference of 0.3 times the standard deviation of the mean between groups at the 5% level of significance. Statistical analysis was performed with SAS software (SAS 9.1.3 Cary, NC).
